# Management of congenital cardiac surgery during COVID-19 pandemic

**DOI:** 10.1017/S1047951120002760

**Published:** 2020-08-24

**Authors:** Atakan Atalay, Başak Soran Türkcan, İrfan Taşoğluİ, Emre Külahçıoğlu, Mustafa Yilmaz, Ata Niyazi Ecevit, Nuri Hakan Aydin

**Affiliations:** Department of Paediatric Cardiovascular Surgery, Ankara City Hospital, Ankara, Turkey

**Keywords:** Paediatric cardiovascular surgery, COVID-19, pandemic paediatric cardiovascular surgery, pandemic

## Abstract

The new coronavirus infection, which was first seen in China in late December, 2019 and eventually became a worldwide pandemic, poses a serious threat to public health. After a high spike in the number of new COVID-19 infection cases following the increase in overall daily death toll in Turkey, Turkish Ministry of Health has taken immediate precautions to postpone elective surgeries in order to reduce the burden to the healthcare system which might be challenged. Whereas different areas of medicine were able to suspend their operative procedures during this period, this was not completely possible in paediatric cardiovascular surgery due to the severity and urgency of congenital heart disease patients requiring operation. Based on the guideline that was published by the Turkish Paediatric Cardiology and Cardiac Surgery Association, in which the patients requiring surgical intervention during the COVID-19 pandemic period are ranked according to the priority, directions were given regarding the operations that hereby, be delayed, we report our experience in 29 cases retrospectively, regarding the pre-operative evaluation of these patients, makings of an emergency operation decision, and strategies taken about intra-operative and post-operative management and arrangements during the pandemic period. In this article, we present crucial precautions that were applied in paediatric cardiovascular surgery and extensive list of cases in order to deliver highest level of the patient safety and protection for the surgical team.

The new coronavirus infection, which started in Wuhan province in China in late December and caused to a worldwide pandemic, poses a serious threat to public health. This new microorganism has been identified as severe acute respiratory syndrome (SARS-CoV-2).

SARS-CoV-2 belongs to a large family of viruses known as coronaviridae. It is known to be a positive-sense single-stranded RNA virus. Other coronaviruses can cause milder illnesses such as flu and much more severe clinical pictures such as the Middle East respiratory syndrome (MERS). SARS-CoV-2 is the 7th coronavirus strain known to infect humans. Other known species are 229 E, NL63, OCU3, HKU1, MERS-CoV, and the original SARS-Cov.^1^

Similar to other coronaviruses, SARS-CoV-2 has four structural proteins known as S (spike), E (envelope), M (membrane), and N (nucleocapsid) proteins. N protein holds the RNA genome, and S, E, and M proteins together form the viral envelope. Spike protein causes the host to adhere to the cell membrane.^2^

The typical symptoms of COVID-19 are fever, sore throat, fatigue, cough, or dyspnoea coupled with recent exposure.^3^ The usual symptoms of COVID-19 include fever (83–98% of all cases), cough (59–82% of all cases), shortness of breath (19–55% of all cases), and muscle ache (11–44% of all cases), which are similar to those of SARS and MERS. Some patients may have sore throat, rhinorrhoea, headache, and confusion a few days before the onset of fever, indicating that fever is a critical symptom, but not the only initial manifestation of infection. The pattern of fever has not yet been fully understood. A small proportion of patients had haemoptysis, and a number of cases were found relatively asymptomatic. COVID-19 patients may have normal or lower white blood cell counts, lymphopenia, or thrombocytopenia, with the increased C-reactive protein level. People who have fever and upper respiratory tract symptoms with leucopenia or lymphopenia should be suspected for this disease, especially for patients with travel history to the endemic area or close exposure record.^4^

A few reports showed that these viruses cause direct myocardial injury mediated via angiotensin converting enzyme-2. Severe hypoxia from acute respiratory damage caused by the virus may also result in oxidative stress and cardiac injury due to increased myocardial oxygen demand.^5^

The virus may cause severe respiratory failure by the massive alveolar damage involving the lower respiratory tract or severe acute respiratory distress syndrome (ARDS). COVID-19 can be fatal, particularly in elderly patients and those with comorbidities. Post-mortem biopsies showed bilateral diffuse alveolar damage with cellular fibromyxoid exudates, interstitial mononuclear inflammatory infiltrates, dominated by lymphocytes, desquamation of pneumocytes, and hyaline membrane formation. The pathological features of COVID-19 much resemble those seen in SARS and MERS coronavirus infection.^6^

On 12 March, 2020, World Health Organisation (WHO) declared this epidemic spread as a pandemic. This outbreak led to 318,763 deaths, with 4,862,329 identified cases worldwide as of 18 May, 2020.^7^

With the COVID-19 pandemic, our national public health infrastructure has faced unpredictable challenges. Planning pandemic management, developing infection prevention policies, and creating algorithms for treatment are important components of this dynamic process. Apart from this, the regulations of the rules for use of personal protective equipment, management of human, and blood resources are other important challenges. In addition, the risk of infection in patients, family members, and healthcare workers is at a considerably high level. Due to the high infection rate of the virus, serious modifications have been made in this process to regulate social life practices. The spread of this pandemic was attempted to be controlled by social isolation.

Another problem faced by the health infrastructure during the pandemic was the efficient use of limited resources. Personal protective equipment has been the most difficult to obtain despite the increasing need in the early days of the pandemic. Against these problems, the re-use of equipment after sterilization has been suggested in some sources.^8^

In line with the increasing unfolding information regarding the SARS-CoV-2 virus, multidisciplinary guidelines have been developed, and these guidelines have been updated frequently.

During the pandemic, babies with congenital heart disease (CHD) continue to be born with an incidence of 1 in 100 live births. About 25% of CHDs are considered critical CHDs needing surgery or other procedures within the first year of life.^9^While many fields in the area of medicine may interrupt their practices during this crisis period, this is not entirely possible due to the severity and urgency of the cases of CHD patients. In particular, a group of newborns and infants often needs surgery in a narrow period of time for an effective result. For this reason, a guideline has been published by the Turkish Paediatric Cardiology and Cardiac Surgery Association. In this guideline, the patients who will require surgical intervention during the COVID-19 pandemic period are ranked according to the priority and directions regarding the operations that can be postponed.^10^

All elective surgical procedures have been cancelled in Ankara by 18 March, 2020 as the first case in Turkey was declared by 11 March, 2020. Our hospital was affirmed as a pandemic hospital after a spike was seen in the number of cases, so our hospital’s resources have been shifted to the management of pandemic. As of this date, 29 congenital cardiac surgery cases have been performed by the paediatric cardiovascular surgery clinic. Our study is a retrospective study regarding the pre-operative evaluation of these patients, makings of an emergency operation decision, and strategies taken about intra-operative and post-operative management and arrangements during the pandemic period.

## Materials and methods

In our study, we report pre-operative evaluation, emergency operation decision making steps, and anaesthesia management of 29 patients who were taken into operation urgently (Table 1). Demographic data, morbidity, and mortality rates were evaluated of those patients whose operations could not be postponed whilst operation strategies in the operating room (OR) and use of personal protective equipment were instated during the COVID-19 pandemic by Ankara City Hospital Paediatric Cardiovascular Surgery between 18 March, 2020 and 18 May, 2020. Transfer to the post-operative intensive care unit and follow-up processes in the intensive care unit were retrospectively analysed.

**Table 1.** Registry of case

Case number	Sex	Age	Weight	Diagnosis	Procedure	Intubation duration (days)	Time in ICU (days)	Mortality (yes/no)
1	Male	3 months	4800 gr	Complete AVSD + CHF	Single patch repair	1	2	No
2	Male	1.5 years	6500 gr	VSD + Severe PH + Reversibility+	VSD closure	0.5	3	No
3	Male	13 years	52 kg	Constrictive pericarditis +CHF+need ECMO support	Total pericardiectomy	1	17	No
4	Male	15 days	3500 gr	Interrupted aortic arch+VSD	Arcus repair+pulmonary banding	8	8	No
5	Female	1.5 years	11 kg	DORV + TGA + VSD + PS	Nikaidoh procedure	4	10	No
6	Male	8 months	7500 gr	TOF + hypoxic spell	Total correction with RV-PA conduit	1	4	No
7	Female	7 years	23 kg	Severe MR + decompensated CHF	MVR	18	18	Yes
8	Female	2 days	2500 gr	Unbalanced AVSD + pulmonary atrezi	4-mm BT shunt	3	3	No
9	Female	4 months	3800 gr	VSD + severe PH + CHF	Correction with pericardial patch	1	4	No
10	Female	11 years	40 kg	Tamponade+cardiogenic Shock	Tamponade revision	0.5	1	No
11	Female	6 days	2900 gr	d-TGA + VSD	JATENE procedure+VSD closure	3	3	Yes
12	Male	15 months	5.500 gr	Complete AVSD + severe PH + trizomy 21	Single patch repair	2	11	No
13	Female	7 days	3000 gr	TOF + pulmonary hypoplasia	4-mm BT shunt	3	3	No
14	Male	7 days	4000 gr	HLVS	Norwood stage 1	8	8	No
15	Male	20 days	3200 gr	d-TGA + VSD	JATENE procedure+VSD closure	6	6	No
16	Female	2.5 months	4000 gr	Swiss cheese VSD + severe PH + KKY	Pulmonary banding+PDA ligation	1	11	No
17	Male	26 days	3500 gr	Truncus arteriosus type 1 + CHF	Rastelli type correction	7	7	No
18	Female	4 days	3200 gr	Ebstein anomaly+pulmonary atresia	4-mm BT shunt	3	3	Yes
19	Male	19 days	3200 gr	Taussig–Bing anomaly+arcus hypoplasia	JATENE procedure+arcus repair	1	1	Yes
20	Male	35 days	1100 gr	Haemodynamically important PDA	PDA ligation	23	Bedside	No
21	Female	26 days	1300 gr	Haemodynamically important PDA	PDA ligation	15	bedside	No
22	Male	13 days	3200 gr	Truncus arteriosus type 1 + CHF	Rastelli type correction	7	7	No
23	Female	5 days	3200 gr	TOF + pulmonary atresia	4-mm BT shunt	14	14	No
24	Female	19 days	3400 gr	HLVS	Norwood stage 1	15	15	No
25	Female	2.5 months	3300 gr	VSD + severe PH + CHF	Pulmonary banding	2	7	No
26	Female	16 days	3000 gr	coarctation of aorta+arcus hypoplasia	Extended side toside anastomosis	2	2	No
27	Female	4 months	5600 gr	VSD + ASD + severe PH + CHF	VSD closure via patch	2	8	No
28	Male	5 months	5000 gr	VSD + ASD + PH + Down syndrome	VSD closure via patch	3	6	No
29	Male	11 days	3300 gr	d-TGA	Jatene	4	4	No

## Results

In total, 16 of the patients who were taken to the emergency operation were neonates (0–28 days) (55.1%), 7 were infants (28 days–1 years) (24.1%), and 6 were children (1–18 years) (20.8%). In total, 14 of the patients (48.2%) were male and 15 were female (51.8%). The average body weight of the patients was 7.560 kg. (min 1.1 kg–max 52 kg). In total, 7 of 16 newborn patients were born in our hospital. Other newborn patients were referred from external centres to our hospital for operation. Twelve newborn patients needed Prostaglandin E1 (PGE1) because of ductus-dependent diseases. After birth, these patients were taken into neonatal intensive care. In total, 2 of the 16 neonatal patients were operated at the neonatal intensive care unit. These patients had very low birth weight and were followed up for patent ductus arteriosus. Therefore, these two were taken into consideration while calculating intensive care periods. The mean duration of neonatal patients hospitalised in paediatric cardiovascular intensive care unit is 6 days (min 1 day–max 15 days). The mean period during which infants and child patients remain intubated is 2.8 days. (min 12 hours–max 18 days), and duration of paediatric intensive care unit stay of these patients is 7.8 days on average (min 1 day–max 18 days). Out of 29 operated patients, 4 patients died (13.8%), of which 3 of them were neonates (75%) and 1 of them was a child (25%). In total, 16 patients (55.1%) were emergent and 13 (44.9%) were in a semi-emergency status.

During this period, the outpatient patients who were admitted to the hospital for operation or referred from external centres were discussed in the council and patients who did not require urgency were informed about the pandemic and the operation of our hospital in this process. Infection risks were explained. The conditions that may develop during the waiting period related to the cardiac diseases were explained. Telephone numbers were given to the patients in case of emergency conditions and operations were postponed. The diagnoses and numbers of patients whose operations were delayed in this 2-month period: Tetralogy of Fallot (TOF) (pink TOF and TOF without history of spell) in 6 patients, complete-partial-intermediate Atrioventricular Septal Defect (AVSD) (mild pulmonary hypertension and no CHF symptoms) in 4 patients, 4 patients with secundum-high venosum Atrial Septal Defect (ASD), and 5 patients with VSD (mild pulmonary hypertension and controlled CHF) were following up with medical therapy.

During the operation planning, the decision was made by the paediatric cardiology – cardiovascular surgery council in the light of the current clinical experience.

## Discussion

### Pre-operative evaluation

Following the evaluation of the patients, born in our hospital or coming from an external centre by the Department of Paediatric Cardiology with the participation of the least number of physicians using videoconference if necessary, operation decisions of the patients were taken at the council. The guideline of Association of Turkish Paediatric Cardiology and Cardiovascular Surgery published on 17 April, 2020 titled “Conducting Paediatric Heart Health services during the COVID-19 pandemic process and cardiac evaluation of infected children.” was followed. According to this source material, the priority order of patients who will require surgical intervention during the COVID-19 pandemic process and the operations that can be postponed are summarised in Tables 2 and 3. However, the operations of newborns with ductus-dependent lesions can be delayed after initiation of the PGE1, and it can lead to new complications day by day. These developing complications may cause newborns to lose their chance of operation. It is therefore critical to determine the optimal time for the operation. In line with this guideline and manuscript, 16 patients (55.1%) were emergent and 13 (44.9%) were in semi-emergency status.^10,11^ However, 2 patients who were taken into operation emergently were not classified based on the guideline. The guideline covered entirely CHDs. There were no interpretations of approach to congenital valve diseases and acquired heart diseases that can be seen in the childhood period. One of these two patients had constrictive pericarditis. The patient was receiving high-dose inotropes due to advanced decompensated heart failure and was followed up in the intensive care unit. Other patient was followed up due to severe mitral insufficiency secondary to acute rheumatic fever carditis. Despite the maximal medical treatment, the left ventricular size increased and the need for diuretics increased. These patients were discussed at the council and it was decided that they needed urgent operation. In addition, there is no classification of childhood cardiac tumours in one of the references source. Although childhood cardiac tumours are rare, mobile or large masses may create acute heart failure or may need urgent surgery due to the risk of embolism. We strongly believe that these groups of diseases should also be included in the current guidelines.

**Table 2.** Sorting the patients who will require surgical intervention in the COVID-19 pandemic according to urgency and the operations that can be postponed.

EMERGENT/EARLY SURGERY Immediate or in 1 to 2 weeks	**SEMI-** ELECTIVE SURGERY In 1 to 3 months	ELECTIVE SURGERY
Drainage of Pericardial Tamponade Ductus dependent newborn with systemic circulation (IAA, HLHS, critical COA, critical AS etc.) Ductus dependent newborn with pulmonary circulation(PA·VSD, PA-IVS, PA-Univentricular heart) Simple cTGA Obstructive TAPVR Severe hypoxic cyanotic CHF Severe newborn with Shone complex Extremely large PDA in preterm Postoperative complication and revision ECMO/assist device need OHT	Non-obstructive TAPVR Large VSD and TGA with PH Truncus arteriosus AP window Complete AV septal defect Tetralogy of Fallot with spell history Functional univentricule requiring pulmonary banding VSD-PH; with CHF unresponsive to medical therapy PDA; baby with PH and heart failure Uncontrolled infective endocarditis High risk cardiac tumor cases Severe LVOT obstruction; symptomatic or LVH HLHS **stage** II	Secundum/sinus venosus ASD closure Partial/intermediate AV septal defect repair VSD closure; normal development, without PH, presence of indication due to extensive shunt or AR Asymptomatic Tetralogy of Fallot Subaortic ridge resection; due to moderate stenosis and mild- moderate AR Glenn operation Completion to Fontan Complex TGA operations without profound hypoxia

AP = aorto-pulmonary; AR = aortic regurgitation; AS = aortic stenosis; AV = atrioventricular; CHF = congestive heart failure; COA = coarctation; ECMO = extracorporeal membrane oxygenation; IAA=“interrupted” aortic arch; LVH=left ventricle hypertrophy; LVOT = left ventricle outflow tract; OHT = orthotopic heart transplantation; PA = pulmonary atresia; PH = pulmonary hypertension; TGA = transposition of great arteries; TAPVR = total anomalous of pulmonary venous return.

*2nd column in the table may vary according to institutes, a multi-disciplinary committee should decide on early operations, taking into account features such as COVID-19 load/density in the hospital and surrounding area as well as whether the hospital is a pandemic hospital

**Table 3.** Congenital lesions and surgical priorities.

For newborns		
Emergent (in 24–48 hours)	Urgent (in 1–2 weeks)	Elective (beyond 2 weeks)
Obstructed TAPVR	TGA with IVS	TGA + VSD
Obstructed cor triatriatum	Symptomatic TOF	Stabile truncus arteriosus
TOF with spell	Ebstein resistant to medical therapy	HLVS
Coarctation unstable with PGE	Coarctation stable with PGE	
Aortic stenosis unstable with PGE	Aortic stenosis stable with PGE	
HLHS with restricted ASD	IVS + PA with PDA(stenting not possible)	
Shunt thrombosis	HLHS	
	Shunt stenosis	
For infants		
Emergent (in 24–48 hours)	Urgent (in 1–2 weeks)	Elective (beyond 2 weeks)
Acute unstable aortic regurgitation	VSD + CHF resistant to medical therapy	VSD + CHF
Prosthetic valve thrombosis	TOF with spell (despite medical therapy)	TOF resistant to medical therapy
Shunt thrombosis	Shunt stenosis	AVSD + trisomy 21 + pulmonary blood overflow resistant to medical therapy requiring surgery
	DCM resistant to medical therapy, restrictive CMP	Ebstein anomaly+ right heart failure
		Mitral insufficiency+CHF
		Symptomatic aortic insufficiency + enlarged left ventricle/decrased LVEF
		Symptomatic Aortis stenosis/LVOTO + decreased LVEF
		RVOTO + impaired right ventricle functions
		Despite shunting, increased cyanosis, or shunt stenosis in bi-directional cavopulmonary anastomosis candidates

For children		
Emergent (in 24–48 hours)	Urgent (in 1–2 weeks)	Elective (beyond 2 weeks)
Acute unstable aortic insufficiency	Worsening CHF with DCM despite medical therapy	Symptomatic heart failure + mitral insufficiency despite medical therapy
Prosthetic valve thrombosis	ARCAPA/ALCAPA + angina with minimal exercise	Aortic insufficiency + decrease in LVEF/LV enlargement
Severe RV dysfunction in patients with RV-PA conduits/conduit stenosis with ventricular arrhythmia	Severe stenosis in RV-PA conduit	Symptomatic AS/LVOTO+ decrease in LVEF
Despite maximal medical therapy, endocarditis in cardiogenic, or septic shock	Stable but uncontrolled endocarditis with ongoing infection	Worsening right heart failure in patients with RV-PA conduits
ARCAPA/ALCAPA+ resuscitated cardiac arrest		Fontan candidates with worsening cyanosis

Based on the second column in the guideline, we made a multi-disciplinary evaluation for our semi-elective patients and decided to perform their operations due to the debilitating effects that may cause patients due to waiting. For instance, pulmonary congestion was present due to pulmonary blood overflow in both patients with truncus arteriosus. Patients were followed up under nasal Continuous Positive Airway Pressure (CPAP) as result of tachypnoea and congestive heart failure. In the case of a 15-month-old patient with VSD, an emergency operation decision was taken at the council due to the positive reversibility test after catheterisation made with the suspicion of Eisenmenger syndrome. During the pre-operative preparations after the decision of the operation was made, any contact with COVID-19-positive individuals, history of travelling abroad, any possible symptoms of COVID-19 were questioned on patients and their close relatives. In the light of current information, there is no clear evidence of vertical transition from placenta. According to the recent data, SARS-CoV-2 virus has not been detected in amniotic fluid, cord blood, or breast milk of pregnant women infected with SARS-CoV-2. The incidence of the disease is lower in neonates than in adults possibly related to the difficulty of vertical transmission of coronavirus, proper handling at birth, and timely isolation of the newborn following the birth. To date, as SARS-CoV-2 has not been detected in breast milk, mothers with COVID-19 can feed breast milk to their babies by the help of uninfected caregivers.^5^ The transition from mother to newborn is thought to be largely due to droplet infection at birth and short-term contact after birth. Therefore, it is important to isolate the baby in need for congenital cardiac surgery born from the COVID-19-positive mother in order to prevent postnatal infection. In our clinic, there was no confirmed COVID-19 positivity in the mothers of newborn patients. Also, when symptom inquiry was made, there was no mother showing symptoms of COVID-19 infection. Seven newborn patients were born in our hospital and immediately separated from their mother due to cyanosis and were taken to intensive care. Therefore, the risk of infection was very low. As 12 of our neonatal patients needed PGE1, they were quickly taken to neonatal intensive care unit after delivery. Two of other 4 patients were taken to the neonatal intensive care unit immediately after birth due to prematurity and had been in intensive care for approximately 1 month. The last 2 patients had congestive heart failure and severe pulmonary hypertension due to pulmonary blood overflow secondary to truncus arteriosus. Therefore, they were followed up in the neonatal intensive care unit. In this way, the isolation of the patients was accomplished. For this reason, these patients were treated more comfortably regarding security measures. However, all preventive measures were strictly followed in patients who came from different hospitals, outside the city, or from abroad. The isolation arrangements made for patients who were taken to the operation from the inpatient ward were, prohibition of visits to floor, limiting the caregiver to the same person and limiting the usage of the common areas with the other patients, imposing the usage of a protective mask for the caregiver, paying utmost attention to hand hygiene, and ensuring that only one patient occupies the room as long as the number of ward beds is suitable.

During this pandemic, many drugs have been recommended by WHO. These medications and treatments are chloroquine, hydroxychloroquine, azithromycin, remdesivir, immunmodulators, and plasma therapy. But none of the medications are not recommended for prophylaxis.

Since hydroxychloroquine may increase the risk of QTc prolongation, extreme care should be taken, especially in patients with CHD. Initial paediatric reports suggest that in the case of severe ARDS, high-dose pulmonary surfactant, inhaled nitric oxide, high-frequency oscillatory ventilation, and extracorporeal membrane oxygenation (ECMO) may be useful.^5^

Only one patient was taken to the intensive care unit following surgery with high dose of inotropic support with ECMO secondary to decreased LV function. In intensive care unit, she was followed up for 18 days with ECMO. The patient had an advanced ARDS picture. Due to COVID-19 suspicion, Polymerase Chain Reaction (PCR) was sent twice from the patient, and both came negative. Gastrointestinal System (GIS) bleeding occurred due to complications regarding ECMO. Bleeding was controlled by platelet replacement and anti-coagulant dose adjustment.

The ARDS emerging from COVID-19 is primarily treated by high flow oxygen therapy, non-invasive mechanical ventilation for eligible patients, mechanical ventilation, and if necessary, putting patient in prone position. In addition, myocardial damage and low cardiac output and arrhythmia may occur due to COVID-19. This condition is called non-coronary myocardial infarction which is thought to be occurring due to hypoxia, excessive inflammatory response, and direct damage of SARS-CoV-2 to cardiomyocytes. In both cases, ECMO is needed. The important point would be which type of ECMO should be used. V-V ECMO in the primary table of ARDS and V-A ECMO in myocardial damage along with cardiogenic shock should be chosen.^6^

Although ECMO temporarily supports cardiorespiratory function, it does not treat the underlying disease. Potential candidates for ECMO must have a reversible cardiorespiratory pathology or be eligible for heart and/or lung transplantation. The use of ECMO is associated with significant risks such as bleeding, infection, frequent transfusion requirements, stroke, microthrombus, or air bubbles passage from the patient line to the circulatory system, and its benefits still contain uncertainty in comparison with conventional treatments. ECMO complications can lead to mortality, morbidity, long-term disability, and a decrease in the quality of life. Mechanical complications include problems with oxygenator, catheter/circuit failure, dysfunctions in the pump or heat exchanger, and problems occurring during cannula insertion and removal. Medical problems related to the patient include bleeding, neurological complications, additional organ failure (e.g., renal, cardiovascular, and liver), barotrauma, infection, and metabolic disorders. Infective complications may be related to intravenous catheters, access sites, or primary pathology. Bleeding is the most common complication in ECMO patients. Bleeding is mostly related to heparin. Also, thrombocytopenia due to ECMO is another factor. The pathogenesis of thromboembolism occurring during ECMO is based on multiple factors such as endothelial activation after contact with the foreign surface, stasis of blood in the heart chambers and veins, and disseminated intravascular coagulation.^12^

A majority of paediatric cardiac surgery operations are performed with cardiopulmonary bypass (CPB). However, this method activates various immunological mechanisms. These mechanisms trigger the formation of systemic inflammatory response syndrome (SIRS) through many pathways such as the complement system, cytokines, coagulation–fibrinolysis cascade, endothelium, and cellular immune system. Surgical stress contact of blood elements with extracorporeal environments, ischaemia/reperfusion injury, hypothermia, and anaesthesia induction are among the causes of SIRS table. This inflammatory process can cause myocardial dysfunction that occurs in the post-operative period, respiratory failure, renal failure, bleeding diathesis, liver dysfunction, and even multi-organ failure.^13^ SIRS is frequently seen in children who have undergone open heart surgery.^14^ COVID-19 also leads to excessive inflammation, oxidation, and exaggerated immune response. The cytokine storm created by this leads to the ARDS table and Multiple Organ Failure (MOF).

### Pre-operative organisation

A separate OR was assigned for COVID-19-positive patients in the OR planning during the pandemic, but operations were not performed in these isolated ORs since there was no positive diagnosis in our patients. However, each patient was considered as a positive case and high levels of protection measures were taken.

Anaesthesia and surgical teams that will perform tracheal intubation and other invasive procedures have taken level 3 protection measures. These measures are consisting of disposable surgical cap, N95/FFP3 mask, scrub, disposable surgical gown, disposable latex gloves, face protective shield, and protective glasses.^15^

The surgeons, anaesthesiologists, anaesthesia technicians, surgical nurses, circulating nurses, perfusionists, and OR staff who comprise of the operation team have been created with the fewest possible people. Thus, contact was minimised. Entries into the OR were restricted once the patient entered in the operating theatre.

During the intubation after anaesthesia induction, the endotracheal tube was clamped and advanced, clamp was released after patient was connected to the ventilator, and not separated from the ventilator during the case. At the end of the case, the endotracheal tube was clamped again before the transport. The patient was taken to the Ambu bag and then the clamp was opened. Thus, transmission risk was reduced.

**Figure 1.**
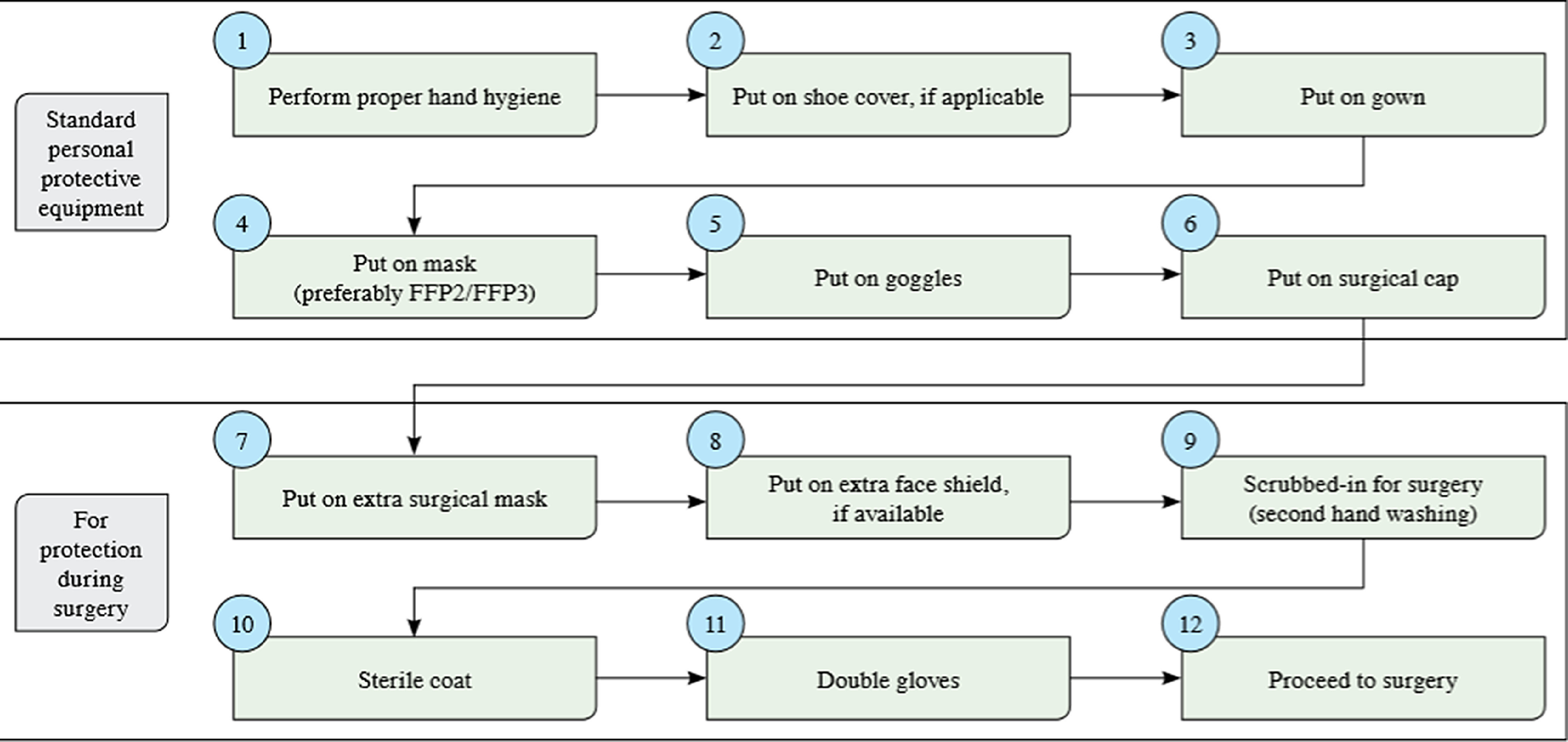
Personal protective equipment during surgery.

Surgeons and surgical nurses wore personal protective equipment in appropriate order before the operation. This sequence is summarised in Table 4 and Figure 1.^16^

**Table 4.** Sequence for donning and doffing personal protective equipment.

Donning procedure	Doffing procedure
Perform proper hand hygiene	Remove shoe covers (if applicable)
Put on shoe cover (if applicable)	Remove gown and gloves together
Put on gown	Perform proper hand hygiene
Put on mask/respirator (if applicable)	Remove eye protection
Put on goggles	Remove mask/respirator (if applicable)
Put on gloves	Perform hand hygiene

One of the difficulties encountered in the pre-operative preparation period was the preparation of blood products. Blood supply has become difficult due to the changes in the blood donation criteria after the outbreak, with the number of donors decreased, the current donation amounts were insufficient to meet the country’s needs, which made donor supply decrease substantially. So, operations of a number of patients had to be postponed multiple times. Besides, another difficulty was the anaesthesia team dealing with congenital heart surgery was assigned to COVID-19 intensive care units. For this reason, case planning was made considering the work schedule and availability of the anaesthesia team.

The resource shortage experienced all over the world is experienced not only in the material but also in blood product resources. Although the virus is not considered to be transmitted by allogeneic blood product transfusion, this is not yet proven. So, patient blood management gains importance in this pandemic process.^15^ The prime solution for CPB is prepared using erythrocyte suspension, fresh frozen plasma, and human albumin, especially in newborns and low-body weight infants, to prevent haemodilution. In this process, we aimed to reduce the patient’s blood product exposure by reducing the amount of blood product used in prime solutions to reduce blood product use. In our clinic, conventional ultrafiltration is routinely used in newborn and low-birth weight infant cases. Throughout the operations performed in this period, excess volume and cytokines were reduced by performing conventional ultrafiltration longer during the CPB and during the separation from the CPB. The use of tranexamic acid, an anti-fibrinolytic that we have already used routinely, continued. Moreover, as reported by Faraoni et al., cut-off haemoglobin values for blood transfusion have been modified both for cyanotic and acyanotic patients to optimise minimum required transfusion amount that patients could tolerate.^17^

At the end of the operation, cases were transferred to paediatric cardiovascular surgery intensive care unit using personal protective equipment. During the transfer, the patient was ventilated by an anaesthesiologist with Ambu bag. The patients were followed up in isolated rooms in the intensive care unit. The doors of the rooms were kept closed, and the nurse giving care during the follow-up entered the patient room using personal protective equipment. This equipment consisted of N95/FF3 masks, gloves, apron, and protective glasses. While entering the patient’s room and after leaving, hands were washed, and hand disinfectants were used.

Neonatal patients were transferred to neonatal intensive care unit after ensuring haemodynamic stability, and inotropic supports were discontinued. In this process, neonatal patients were followed up intubated in paediatric cardiovascular intensive care unit. Therefore, the intubation times in the table are the same as the time they stay in the intensive care unit of paediatric cardiovascular surgery. After the neonatal patients were transferred to the neonatal intensive care unit, the post-operative follow-up of the patients continued.

### Post-operative management

In the post-operative period, four patients were suspected of coronavirus infection due to lung infiltration in imaging and treatment of resistant fever. Chest x-rays of two patients were confirmatory with ARDS. PCR tests were sent for both patients, but results were negative. However, since the false negativity rates cannot be ignored, these patients were evaluated as positive and preventive measures were taken in intensive care unit. Two of these 4 patients were followed up as intubated when the test was sent. Peak pressure of 30–35 cmH2O and tidal volume of 4–8 ml/kg were adjusted in patients with ARDS. For mechanical ventilator mode, although there was no significant difference between volume-controlled and pressure-controlled modes, we preferred pressure-controlled mode to adjust peak pressure more efficiently. During ventilation, high PaCO2 values were aimed, such as controlled respiratory acidosis, and not modified ventilatory settings unless the pH drops below 7.20–7.25. Initially, adjustments were made for the FiO2 set to 1, and gradually lowered to 0.6 as long as PaO2 was greater than 60 mmHg and/or oxygen saturation was 90%.^14,18^

There are different methods in cardiac surgery that reduce this inflammatory process. Surgery performed in moderate hypothermia in some patients, modified or conventional ultrafiltration, heparin-coated circuits, short-CPB times, and glucocorticoids are some of these methods.

In paediatric cardiac surgery patients, glucocorticoids are used prophylactically to reduce the SIRS response due to CPB, but in general, COVID-19 is not recommended due to the possibility of prolonging viral replication time. Its use in this process should be used considering the benefit–loss ratio. Since there was no suspicion of COVID-19 in our operated patients, so that we continued routine use of 5 mg/kg of methylprednisolone in cases with CPB, importantly this entity is a condition that cannot be ignored in this process.^19^

Infant and child patients were extubated at the earliest possible period, bearing in mind their haemodynamic parameters. In total, 3 of 13 patients were required re-intubation. During these tracheal intubations performed by experienced anaesthesiologists, the team conducting intervention used personal protective equipment and all steps applied in OR for intubation were followed.

The patients were taken to the ward after the haemodynamic stability was achieved and the need for intensive care was ended. Chest drainage tubes, urinary catheters, and invasive arterial catheters were withdrawn in intensive care unit before being taken into ward and were taken into single rooms. Visitors are prohibited from entering the floor, and caregivers were asked not to be changed as much as possible. Social isolation rules were strictly applied in the common areas.

The mortality in the operations we perform in this process is 13.8%. It is relatively high compared to our overall ratio before pandemic. We believe the reasons for this elevation are performing only emergency and urgent operations and due to reduced resources during the pandemic period. For example, transfer of teams experienced in congenital cardiac surgery to COVID-19 intensive care units, transfer of experienced nurses working in paediatric cardiovascular surgery intensive care units to other departments, and problems with supply of blood products were all contributing factors.

## Conclusion

Due to the pandemic caused by the SARS-CoV-2 virus, which started at the end of December in Wuhan province of China and resulted with a worldwide pandemic, many fields have interrupted their studies and shifted their resources to the pandemic management. It is not possible to interrupt paediatric cardiac surgery due to high volume of emergency cases and limited time interval for newborns who need surgery in a narrow window of time. For this reason, in the event of a crisis, it is vital that the limited resources are directed efficiently, and operations should be continued. In this process, emergency surgery case selection is extremely critical. Although this choice is updated according to the literature, we think that childhood-acquired valvular diseases, cardiac tumours, and pericarditis should also be included in the guidelines in line with the increasing knowledge. In this process, the surgeon, anaesthesiologist, nurse, perfusionist, and support team should continue their practices by being protected from the pandemic. Although transfusion-related contamination has not been proven, supply of blood products, which is one of the biggest obstacles in the process, should be increased by analysing the risks and doing the necessary donor evaluations. This process should also be promoted by increasing the social awareness about the vitality of blood donation even during pandemic times.

## References

[1] Zhu N , Zhang D , Wang W , et al. A novel coronavirus from patients with pneumonia in China, 2019. N Engl J Med 2020; 382: 727–733. doi: 10.1056/NEJMoa2001017. 31978945 PMC7092803

[2] Wu C , Liu Y , Yang Y , et al. Analysis of therapeutic targets for SARS-CoV-2 and discovery of potential drugs by computational methods. Acta Pharm Sin B 2020; 10: 766–788. doi: 10.1016/j.apsb.2020.02.008. 32292689 PMC7102550

[3] Zhai P , Ding Y , Wu X , et al. The epidemiology, diagnosis and treatment of COVID-19. Int J Antimicrob Ag. doi: 10.1016/j.ijantimicag.2020.105955. PMC7138178 32234468

[4] Tu Y-F , Chien C-S , Yarmishyn AA , et al A review of SARS-CoV-2 and the ongoing Clinical Trials. Int J Mol Sci 2020; 21: 2657 10.3390/ijms21072657 PMC7177898 32290293

[5] Dilli D , Tasoglu I. Perioperative care of the newborns with congenital heart diseases in the time of COVID-19. doi: 10.1017/S1047951120001845. PMC7322211 32613934

[6] Akar AR , Ertugay S , Kervan Ü , et al Turkish Society of Cardiovascular Surgery (TSCVS) Proposal for use of ECMO in respiratory and circulatory failure in COVID-19 pandemic era. Turk Gogus Kalp Dama 2020; 28: 229–235. 10.5606/tgkdc.dergisi.2020.09293 PMC7298378 32551150

[7] https://www.worldometers.info/coronavirus/.

[8] Mavioglu HL , Unal EU. Cardiovascular surgery in the COVID-19 pandemic. J Card Surg 2020; 35: 1391. 32306474 10.1111/jocs.14559 PMC7264533

[9] Dolk H , Loane M , Garne E. Congenital heart defects in Europe: prevalence and perinatal mortality, 2000 to 2005. Circulation 2011; 123: 841–849. 21321151 10.1161/CIRCULATIONAHA.110.958405

[10] https://turkpedkar.org.tr/tr/covid-19-ve-cocuk-kalp-sagligi-rehberi/.

[11] Stephens EH , Dearani JA , Gulesarian KJ , et al. COVID-19: crisis management in congenital heart surgery. JThorac Cardiovasc Surg 2020. doi: 10.1016/j.jtcvs.2020.04.006. PMC7307004 32496871

[12] Akarsu Ayazoğlu ve ark. Erişkin ARDS Hastalarında ECMO. J Turk Soc Intensive Care 2015; 13: 95106

[13] Araz C , Pirat A . Koroner Arter Bypass Cerrahisinde Kardiyopulmoner Bypass İlişkili Erken Dönem İnflamatuar Yanıt ve Yoğun Bakım Kalış Süresi Üzerine Atorvastatinin Etkileri. J Turk Soc Intens Care 2011; 9.

[14] Durandy Y . Minimizing systemic inflammation during cardiopulmonary bypass in the pediatric population. Artif Organs 2014; 38: 11–18. doi: 10.1111/aor.12195. 24392866

[15] Günaydın S. Perioperative planning in the Covid-19 pandemic: cardiovascular perfussion and devicerelated issuses. Turk Gogus Kalp Dama 2020; 28: 247–249. 10.5606/tgkdc.dergisi.2020.09296 PMC7298380 32551153

[16] Mavioğlu HL , Ünal EU , Aşkın G , Küçüker ŞA , Özatik MA. Perioperative planning for cardiovascular operations in the COVID-19 pandemic. Turk Gogus Kalp Dama 2020; 28: 236–243. 10.5606/tgkdc.dergisi.2020.09294 PMC7298362 32551151

[17] Faraoni D , Meier J , New HV , Van der Linden PJ , Hunt BJ. Patient blood management for neonates and children undergoing cardiac surgery: 2019 NATA guidelines. J Cardiothorac Vasc Anesth 2019; 33: 3249–3263. 31076306 10.1053/j.jvca.2019.03.036

[18] Günen H , Kizkin Ö . ARDS’de mekanik ventilasyon prensipleri. Tüberküloz ve Toraks Dergisi 2004; 52: 199–206. 15241708

[19] Townsley MM , Crawford JH . Steroids for pediatric cardiac surgery: can we put the discussion to rest?. J Cardiothorac Vasc Anesth 2020; 34: 1548–1549. 31837962 10.1053/j.jvca.2019.11.024

